# Occupational stress and job dissatisfaction with health work

**DOI:** 10.1186/s41155-019-0132-5

**Published:** 2019-09-23

**Authors:** Camila Carvalho de Sousa, Tânia Maria de Araújo, Iracema Lua, Mariana Rabelo Gomes

**Affiliations:** 1 Núcleo de Epidemiologia, State University of Feira de Santana, Av. Transnordestina, s/n, Novo Horizonte, Feira de Santana, Bahia 44036-900 Brazil; 2 College of Medicine of Bahia, Federal University of Bahia, Largo do Terreiro de Jesus, s/n, Centro Histórico, Salvador, Bahia 40026-010 Brazil

**Keywords:** Psychosocial aspects at work, Occupational stress, Job satisfaction, Health workers, Job strain model, Effort-reward imbalance model

## Abstract

**Objective:**

The purpose of this study is to evaluate the association between psychosocial aspects at work and dissatisfaction among health workers in five cities in Bahia, Brazil.

**Methods:**

The evaluation was based on different models proposed to measuring occupational stress and possible combinations between them: demand-control model (DCM) and effort-reward imbalance (ERI). We conducted a cross-sectional epidemiological study including 3084 health workers. The analysis considered the association between partial/full/partial (combined) occupational stress models (the variable “exposure”) and job dissatisfaction (the variable “outcome”).

**Results:**

Dissatisfaction rate was 26%. Full DCM and ERI models were better than partial ones to investigate job dissatisfaction. After adjustments, the combined models presented more robust measures of prevalence ratio than models evaluated separately (PR 2.93; CI 2.26–3.80).

**Conclusions:**

The combination of models has shown greater capacity to identify situations of job dissatisfaction and provided more potential information to support actions for workers’ health.

## Background

High levels of occupational stress may occur in several ways among health workers, and job dissatisfaction is one of them (de Oliveira, Silva, Galvão, & Lopes, 2018; Fila, Paik, Griffeth, & Allen, 2014; Hosseinabadi et al., 2018). Considered as an unpleasant emotional state from worker’s appraisal of his/her job, or because the job is not according to his/her code of values, job dissatisfaction is closely related to psychosocial aspects at work (Ribeiro, Assunção, & Araújo, 2014).

Psychosocial aspects can directly influence the job dissatisfaction levels because such levels are based on the worker’s experiences, judgments, and appraisals of these aspects (de Oliveira et al., 2018). Thus, job dissatisfaction results from job elements and can be defined as a complex interaction of tasks, responsibilities, incentives, and rewards in a given context (Martinez, Paraguay, & Latorre, 2004).

In this study, we focus on job dissatisfaction among health workers. Current characteristics of work in health services, in line with global changes in the world of work, have been associated with several adverse outcomes in the health and quality of life of this professional group (Assunção, 2011). They are inserted in a scenario characterized by increased precariousness of work, reduction of formal work, loss of labor rights, and marked wage inequality between men and women (ILO, 2016, 2017). It has been observed that the adoption of neoliberal policies in the countries of Latin America has favored the instability and insecurity of employment, with high labor exploitation (Machado, Giongo, & Mendes, 2016).

In this context, Brazilian workers, particularly health workers, have been experiencing increasing levels of flexibilization of employment bond and loss of labor rights in recent years. In this sense, these workers, in order to guarantee their jobs, are subject to work overload, double shift, precarious conditions, low remuneration, and increase in the exposure to work accidents, with repercussions on the physical and mental health (Maciel, Santos, & Rodrigues, 2015) and, consequently, on the levels of satisfaction with work.

Having a job does not ensure professional and personal fulfillment as the work environment is not neutral and brings many situations related to the organization and division of labor. Although these situations can produce feelings of pleasure, self-worth, and well-being, they can bring discomfort, low esteem, sadness, and devaluation, thus resulting in high levels of satisfaction or dissatisfaction. Therefore, analyzing the work aspects that can produce both effects is a relevant task as satisfaction/dissatisfaction can lead to suffering and illness. Psychosocial aspects at work are relevant to these outcomes (Martinez et al., 2004). Therefore, monitoring these aspects of work environments can be useful for work management in establishing potential measures to improve the conditions offered.

Different instruments for measuring the psychosocial aspects at work have been developed such as the demand-control model (DCM) proposed by Karasek (1979, 2008) and the ERI (effort-reward imbalance) model elaborated by Siegrist (1996). Both are widely used in the national and international literature because they perform well in assessing the psychosocial characteristics of work and their outcomes to the worker’s health. In this study, the effects of work psychosocial aspects on job dissatisfaction will be analyzed, considering job dissatisfaction as an important mediator of suffering and mental illness among health workers (Ribeiro et al., 2014).

The demand-control model highlights two central elements at work: (1) the psychological demands related to psychological requirements faced by the health worker during the execution of his/her work activities (e.g., work pace, concentration level required, interruption of tasks) and (2) worker’s control over activities performed, including the autonomy in the decision-making process. Social support constitutes a third dimension later included in the DCM by Johnson and Hall (1988). The hypothesis is this dimension can minimize damages to the worker’s health caused by high job demands and low control over the work itself. According to the authors, colleagues and superiors who support the accomplishment of the tasks, social integration, and confidence in the group can be protectors against work-related health strain from the workspace.

The Job Content Questionnaire (JCQ) is an instrument proposed to measure these job strain dimensions. The JCQ performed well to investigate psychosocial aspects at work among Brazilian workers in previous studies (Araújo & Karasek, 2008).

The effort-reward imbalance (ERI) model was based on the reciprocity of social relations in the work environment. According to the model, efforts are equalized by respective rewards. The imbalance between efforts expended on the job and the rewards received results in job stress. High efforts followed by low rewards are likely to trigger negative emotions and sustained stress responses, while the balance between effort and reward generates positive emotions capable of promoting well-being and health at work (Siegrist, 1996). Effort and reward are extrinsic components of this model.

The ERI model, as well as DCM, also has a third dimension: the overcommitment. This is an intrinsic component of the model. This dimension is characterized as a worker’s excessive effort for recognition and approval. The interaction between high overcommitment and effort-reward imbalance is harmful to the workers and exposes them to high stress levels (Jonge, Bosma, Peter, & Siegrist, 2000; Siegrist & Wahrendorf, 2016).

These models have been extensively tested in several developed countries, with emphasis on European countries, Canada, Japan, and the USA (Alves, Hökerberg, & Faerstein, 2013; Siegrist & Wahrendorf, 2016). More recently, studies in Brazil have also used these models. Additionally, studies have shown the use of combined models for investigating outcomes of the worker’s health as each model addresses a specific set of occupational stressors (Araújo, Mattos, MMG, & Santos, 2016; Griep, Rotenberg, Landsbergis, & Vasconcellos-Silva, 2011; Pinhatti et al., 2018; Yu et al., 2013). This is an attempt to minimize the limitations of the isolated models to understand the work environment complexity. Based on the assumption that different exposures in each of these models allow us to overcome the limitations of the isolated models, the purpose of this study was to evaluate the association between the psychosocial aspects at work and job dissatisfaction for health workers based on different models (partial and full ones) and possible combinations of them (DCM and ERI).

It is expected that the exploration of this subject may contribute to produce knowledge in an area still little explored in Brazil, the association among occupational stress and effects on job dissatisfaction.

## Methods

This is a cross-sectional epidemiological study conducted in five municipalities in the state of Bahia in 2011 and 2012, with health workers from medium complexity/primary care services.

The study population was defined by a previous survey of the workers at the Municipal Health Secretariats of the five municipalities, where the study took place. The number and type of health services available, number of employees, and their occupations, as well as the geographic area in which each service was located, were delimited. Based on the list of all workers and estimates of health outcomes, the sample size of the original study was defined (based on the outcome that resulted in the largest sample size). After this, a representative sample of health workers was obtained by random and stratified sampling, and proportionally organized per geographical areas (coverage areas of the Family Health Care units), complexity level in the health services network (primary or medium complexity care), and professional category. The sampling population comprised all previously identified health workers of each municipality. Then, the randomly selected workers were contacted at their respective work environments and invited to be part of the study.

Inclusion criteria were the following: workers who were in effective professional practice, who accepted to participate in the study, and who had been working for at least 6 months at the health unit. We try to find the raffled workers and conduct the interview by three attempts. Those who refused to participate in the study, those who were away from their work in the period of data collection, or those who were not found in the three attempts were replaced with respect to the geographical area, level of complexity of the service, group occupation, and sex.

Considering that the sample had not been calculated for the purpose of this study, in order to verify whether the multicenter study had the power to evaluate the association between work psychosocial aspects and dissatisfaction, the sample was recalculated using job dissatisfaction as the outcome of interest. Such calculation was performed using the software OpenEpi, version 3.03^a^. Based on the total population of health workers in the five municipalities (*N* = 4278), the prevalence of the event of interest was at 44.9% (Fadel, Carvalho, Arcieri, Saliba, & Garbin, 2008), confidence interval at 95.0%, absolute error at 2%, and estimate rate for losses/refusals at 20%. Finally, the estimated number of the sample was 1834 workers. A total of 3084 workers were investigated, thus providing the study with sufficient power for the intended analysis.

Data collection has used a structured questionnaire, based on the literature review, previously tested in a pilot study. In order to standardize the methodological procedures adopted at each location, a manual of procedures was prepared and workshops were held to train and prepare the interviewers.

Job dissatisfaction was assessed by the following question of the Job Content Questionnaire (JCQ): Are you satisfied with your job? Response options included a Likert scale with score from 1 to 4 with the following response options: 1, I am not satisfied at all; 2, I am not satisfied; 3, I am satisfied; and 4, I am very satisfied. For the purposes of analysis, the variable was dichotomized in satisfied (answers 3 and 4) and dissatisfied (answers 1 and 2).

This type of scale to evaluate dissatisfaction is feasible because satisfaction and dissatisfaction represent opposite meanings of the same phenomenon. Satisfaction with work is caused by multiple factors, and this multicausality of the events must be considered. However, studies have demonstrated the feasibility of measuring job satisfaction with a single question. These approaches have shown a positive correlation of measurements made from multidimensional (multiquestions) questionnaires. In addition, one question is more sensitive to capture variations in job dissatisfaction (Ommen et al., 2009; Wanous, Reichers, & Hudy, 1997).

The two main exposure variables were considered as follows: the “demand-control model” measured by the JCQ and the “effort-reward imbalance” obtained from the ERI scale.

The DCM variable was measured by combining job aspects, psychological demand, and control, and the median was considered as a cutoff point for defining categories as high and low. Based on these levels, four specific work situations have been established and considered various risks to worker’s health as follows : low strain (high control/low demand), active job (high control/high demand), passive job (low control/low demand), and high strain (low control/high demand) (Araújo & Karasek, 2008). DCM score was calculated from the ratio between demand and control (D/C) dimensions in order to make DCM comparable to ERI and, thus, combine these two models. By using the mean as a cutoff point, workers were classified as exposed and non-exposed, based on the dichotomous categorization of the scores obtained (values ≤ mean = not exposed, values > mean = exposed) (Griep et al., 2011).

The ERI model was developed from a self-administered questionnaire with Likert-scale responses (1, “I strongly disagree”; 2, “I disagree”; 3, “I agree”; 4, “I strongly agree”). The mean was also used as a cutoff point for defining the proposed scales in this model: effort (high/low), reward (high/low), and overcommitment (present/absent). The effort-reward imbalance indicator was obtained by the following formula: ERI = *e*/(*r* × *c*), where *e* refers to the sum of the effort items, *r* is the sum of the reward items, and *c* is a correction factor. The results were categorized as “balance” (values ≤ 1) and “imbalance” (values > 1)^7^. Here, workers were classified as exposed and not exposed using 1 as the baseline value (values ≤ 1 = not exposed, values > 1 = exposed) (Griep et al., 2011).

The analysis considered the association between job dissatisfaction and the partial, full, and partial (combined) occupational stress models. Thus, the following five models were evaluated: partial DCM (psychological demand and control over one’s own work), full DCM (demand-control and “social support” in the work), partial ERI (effort and reward), full ERI (including the dimension “overcommitment”), and the combined models (partial DCM combined with partial ERI).

The following covariables, known to be related to job dissatisfaction, were also included in the analyses: socio-demographic characteristics (sex, age, skin color, marital status, children, and income) and professional information (professional practicing time, employment bond, work shift, compatibility with the job description, weekly working hours, having another job, and having labor rights, like vacation and thirteenth salary) based on the literature (Carrillo-Garcia et al., 2013; Lapischies, Jardim, & Kantorski, 2014; Ribeiro et al., 2014; Tambasco, da Silva, Pinheiro, & Gutierrez, 2017).

For the statistical analysis of the data, the studied population was initially described, followed by bivariate, stratified, and multivariate analyses. The bivariate analysis calculated the prevalence ratios (PR) and 95% confidence intervals (CI). Also, *p* values were calculated using Pearson’s chi-square test to evaluate the statistical significance of the associations. The Statistical Program for Social Sciences 24.0 (SPSS 24.0) and OpenEpi 3.0 were used in this phase.

In order to investigate confounding factors, variations equal to or greater than 20% between crude and adjusted associations per each covariable of interest, as well as theoretical evidences, were considered. As none of the covariables investigated has varied greater than 20%, to select the confounding variables, we applied the theoretical knowledge based on literature review. These variables were added to adjust the final model.

The logistic regression was applied in the multivariate analysis, which generates odds ratio (OR) as a measure of association. Also, the robust Poisson regression method was used to properly estimate the prevalence ratios (PRs) and their respective 95% confidence intervals (Coutinho, Scazufca, & Menezes, 2008; Francisco et al., 2008). The software STATA 12.0 was used in this step.

The final model’s quality assurance and fit model were assessed by the goodness-of-fit test (Hosmer and Lemeshow), ROC curve analysis, and pattern of influential observations.

The regulatory ethical resolution 466/2012 of the Brazilian National Health Council was complied with at all stages of the research, and the study was approved by the Ethical Institutional Review Board (IRB) under the protocol number 081/2009 (CAE 0086.0.059.000. -09).

## Results

The sample consisted of 3084 health workers, predominantly women (78.2%), young workers up to 35 years old (42.0%), and between 36 and 50 years old (39.5%). The predominant skin color was black or Brazilian *pardo* (mixed race) (82.8%). More than half (55.1%) of the population has a primary or secondary level of education, lives with a partner (57.3%), and reports having children (68.5%). The prevailing monthly income is up to two minimum wages (70.8%).

Just over half of the workers have been in the profession for more than 5 years (56.9%) and had a stable employment (64.8%). Most reported working during daytime shifts (79.8%), in functions compatible with the job description (93.7%), and having at least one labor right (94.5%). Approximately three quarters of the sample have reported having only one job (74.8%) and a working day of up to 40 weekly hours (79.1%).

Regarding work psychosocial aspects, 48.9% reported low control over work, 43.4% reported high psychological demand, and 71.3% had low social support. With regard to quadrants of the DCM, 70.7% of workers had experienced occupational stress situations (27.1% in passive work, 21.9% in active work, and 21.7% in high strain situations) (Table 1).

**Table 1:** Dimensions of the demand-control model. Health workers, Bahia, Brazil, 2012

Dimensions of the DCM	Number	Percentage
Control over work (*n* = 2936)		
Low	1437	48.9
High	1499	51.1
Psychological demand (*n* = 3025)		
Low	1713	56.6
High	1312	43.4
Social support (*n* = 2864)		
Low	2043	71.3
High	821	28.7
Demand-control model (*n* = 2890)		
Low strain requirement	846	29.3
Passive work	784	27.1
Active work	629	21.9
High strain	631	21.7

Considering the dimensions of the ERI model, 31.8% had reported high effort, 69.8% low reward, and 45.2% high overcommitment to work. It is worth noting the high amount of workers in an effort-reward imbalance situation (68.8%) (Table 2).

**Table 2:** Dimensions of the effort-reward imbalance model. Health workers, Bahia, Brazil, 2012

Dimensions of the ERI	Number	Percentage
Effort (*n* = 3048)		
Low	2096	68.8
High	952	31.2
Reward (*n* = 3014)		
Low	2105	69.8
High	909	30.2
Overcommitment (*n* = 3040)		
Absent	1666	54.8
Present	1374	45.2
Effort-reward imbalance model (*n* = 2987)		
Balance	933	31.2
Imbalance	2054	68.8

The job dissatisfaction rate was at 26%. The following situations were related to dissatisfaction at statistically significant levels: low control over work (adjusted PR 1.82; CI 1.57–2.11), high psychological demand (adjusted PR 1.56; CI 1.36–1.79), and low social support (adjusted PR 1.77; CI 1.47–2.13). Exposure to occupational stressors (high demand or low control), either alone (passive or active work) or combined (work in high demand), was statistically associated with job dissatisfaction (Table 3), even after adjusting per gender, educational level, income, employment bond, and activities compatible with the job description. The highest association was found in the high strain situation, which is almost three times higher in this group than the reference category (low strain).

**Table 3:** Job dissatisfaction according to the demand-control model among health workers, Bahia, Brazil, 2012

Model scales	Job dissatisfaction					
	*n*	*P* (%)	PR	CI (95%)	PR*	CI (95%)*
Control						
Low	490	34.2	1.83	1.62–2.09	1.82	1.57–2.11
High	278	18.6	*		*	
Psychological demand						
Low	358	20.9	*		*	
High	429	32.8	1.56	1.39–1.77	1.56	1.36–1.79
Social support						
Low	610	29.9	1.76	1.49–2.08	1.77	1.47–2.13
High	139	17.0	*			
Demand-control model						
Low strain	123	14.6	*		*	
Passive work	223	28.5	1.96	1.60–2.38	1.99	1.59–2.49
Active work	151	24.0	1.65	1.33–2.04	1.74	1.37–2.21
High strain	260	41.4	2.84	2.36–3.43	2.81	2.78–3.48

^*^Adjustment for gender, educational level, income, employment bond, and activities compatible with the position

The dimensions of the ERI model were associated with job dissatisfaction at statistically significant levels: high effort (adjusted PR 1.66; CI 1.45–1.89), overcommitment to work (adjusted PR 1.42; CI 1.24–1.63), and effort-reward imbalance (adjusted PR 1.73; CI 1.44–2.08), even after adjusting per gender, educational level, income, employment bond, and activities compatible with the job description (Table 4).

**Table 4:** Job dissatisfaction according to effort-reward imbalance model. Health workers, Bahia, Brazil, 2012

Exposure types	Job dissatisfaction					
	*n*	*P* (%)	PR	CI (95%)	PR*	CI (95%)*
Effort						
Low	448	21.4	*		*	
High	343	36.0	1.68	1.46–1.89	1.66	1.45–1.89
Reward						
Low	567	27.0	1.11	0.97–1.27	1.16	0.99–1.36
High	221	24.3	*		*	
Overcommitment						
Absent	353	21.2	*		*	
Present	442	32.2	1.52	1.35–.71	1.42	1.24–1.63
Effort-reward imbalance model						
Balance	161	17.3	*		*	
Imbalance	618	30.1	1.75	1.49–2.04	1.73	1.44–2.08

*Adjustment for gender, educational level, income, employment bond, and activities compatible with the position

By evaluating the full demand-control model and social support, it was observed that both exposures were associated with job dissatisfaction when considered separately. However, when analyzed together, a higher power of association was found (adjusted PR 2.70, CI 2.10–3.47) (Table 5).

**Table 5:** Job dissatisfaction by partial, full, and combined occupational stress models. Health workers, Bahia, Brazil, 2012

Exposure types	Job dissatisfaction					
	*n*	*P* (%)	PR	CI (95%)	PR*	CI (95%)*
Demand-control (DC) model and social support at work						
DC and social support without exposure	73	13.8	*		*	
Exposure in DC	64	26.2	1.89	1.41–2.56	1.80	1.29–2.52
Exposure in social support	224	22.9	1.66	1.30–2.11	1.60	1.23–2.09
Exposure in DC and social support	355	37.4	2.70	2.15–3.96	2.70	2.10–3.47
Effort-reward imbalance (ERI) and overcommitment (OC)						
ERI and OC without exposure	109	15.2	*		*	
Exposure in ERI	239	26.5	1.74	1.42–2.14	1.69	1.33–2.13
Exposure in OC	51	24.8	1.63	1.2–2.19	1.37	0.95–1.96
Exposure in ERI and OC	376	33.2	2.19	1.8–2.65	2.05	1.65–2.56
Combined models						
DC and ERI without exposure	84	14.1	*		*	
Exposure only in DC	74	25.8	1.82	1.38–2.41	2.17	1.56–3.01
Exposure only in ERI	221	22.4	1.59	1.26–1.99	1.75	1.33–2.30
Exposure in DC and ERI	364	38.2	2.69	2.18–3.34	2.93	2.26–3.80

^*^Adjustment for gender, educational level, income, employment bond, and activities compatible with the position

The isolated ERI model was associated with job dissatisfaction at statistically significant levels; this was not observed for the overcommitment component. Despite being associated with dissatisfaction, overcommitment alone did not reach statistical significance when adjusted by the covariables. However, when the full model (ERI + overcommitment) was considered, the power of the association has increased and statistical significance was obtained (adjusted PR 2.05, CI 1.65–2.56). This has shown that the dissatisfaction rate has increased in situations where both exposures were present.

In the comparative analysis of dissatisfaction by considering the DCM and ERI models, it was observed that the DCM groups have shown a measure of association greater than the ERI ones. However, combining DCM and ERI models results in the greatest power of association (adjusted PR 2.93; CI 2.26–3.80) among all the analyses performed, after adjusting the model per the covariables gender, educational level, employment bond, and activities compatible with the position. Therefore, the combined use of occupational stress models was the most advantageous to identify job dissatisfaction situations.

The study concluded that the models were well suited to the data (Hosmer and Lemeshow test presented *p* values > 0.05 in all three models). The ROC curve showed an area equal to 0.669 (partial and full DCM), 0.646 (partial and full ERI), and 0.672 (partial models combined).

Then, the pattern of covariables was analyzed to evaluate if extreme observations found were influential. The comparison of the models, with and without the observations, has shown little influence, and there was no significant change in the Hosmer and Lemeshow test and the ROC curve.

## Discussion

A high feminization of health professions was found according to the profile of workers, who are relatively young, and these factors may influence job dissatisfaction as already evidenced in other studies (Carrillo-Garcia et al., 2013). In addition, there are psychosocial aspects at work, which are acknowledged sources of stress and dissatisfaction (de Oliveira et al., 2018; Hosseinabadi et al., 2018; Ribeiro et al., 2014).

The job dissatisfaction levels, although lower than those found in other studies with similar occupational groups (Fadel et al., 2008; Fernandes, Miranzi, Iwamoto, Tavares, & Santos, 2012; Ribeiro et al., 2014), is an alarming indicator. It turns out that approximately one of four workers was dissatisfied with their work. Considering that high levels of dissatisfaction can result in physical and mental exhaustion, while contributing to low self-esteem and loss of interest, and triggering adverse behaviors such as irritability and bad mood—harmful to the health of the worker (Melo, Barbosa, & Souza, 2011), this population should be considered at risk of becoming ill and requires workplace intervention and follow-up.

The job dissatisfaction was associated with the dimensions of the partial, full, and combined DC and ERI models. DCM dimensions were strongly associated with dissatisfaction at statistically significant levels, where the dimension “control over work” had the greatest power of association with the outcome, followed by the dimensions of “social support” and “psychological demands,” respectively. High strain, passive work, and active work, in this order, were directly associated with dissatisfaction with increasing levels of prevalence ratios, when compared to the low strain group.

Control over one’s own work refers to the worker’s freedom to choose how to act, based on the skills of the worker. However, considering the dynamics of health work, new rules have frequently been implemented and straining workers increasingly. In order to maintain a certain degree of psychological balance, the strategies previously used by the worker to adapt to the labor demands are sometimes ineffective because the new imposed routines may result in imbalance and stress (Karasek, 2008). Thus, low levels of autonomy at work expose the worker to stress and generate job dissatisfaction (Ribeiro et al., 2014).

These findings are similar to those reported by Ribeiro et al. (2014), in a study carried out with Brazilian physicians, and by Elliott, Rodwell, and Martin (2017), in a study with nurses in Australia. Both have shown a positive relationship between low control and job dissatisfaction, where the negative perception of self-control over one’s work likely leads to dissatisfaction.

The highest level of psychological demand referred by workers was associated with dissatisfaction. This reinforces the hypothesis that high levels of psychological demand predispose workers to job dissatisfaction (Ferreira, 2015; Hosseinabadi et al., 2018; Soratto et al., 2017). The balance required by the individual to perform tasks and maintain psychic integrity is impaired by increased demand, resulting in occupational stress (Karasek, 2008). High demand can trigger work acceleration by preventing work from being done as it should. This may impact care quality and resolution, which lessens the experience that the work has achieved its purposes.

High psychological demand along with low control over work were associated with high job dissatisfaction levels, which exposes workers to frequent psychological loads increasing emotional exhaustion and health problems. This association was evidenced in other studies (Elliott et al., 2017; Fernandes et al., 2012; Hosseinabadi et al., 2018; Jonge et al., 2000).

Social support is capable of minimizing the adverse effects of the work environment (Mattos, Araújo, & Almeida, 2017), and it is paramount for maintaining high levels of satisfaction among workers. Developing team activities is one of the most significant reasons for job satisfaction among health workers. Taking into account common goals, collective and shared work among a multidisciplinary team can lead to increase in the efficiency of services, thus generating greater workers’ satisfaction (Lima, Pires, Forte, & Medeiros, 2014). As health work is always teamwork, the perception of support can define personal situations of esteem, safety, and well-being. Thus, this work dimension is important to worker’s satisfaction.

Considering ERI, the dimension “effort” was the most associated with dissatisfaction, followed by “overcommitment.” The dimension “reward” was associated with job dissatisfaction but did not reach statistical significance. However, the effort-reward imbalance almost doubled the job dissatisfaction rate.

When the work effort does not match the rewards received, that is, when there is an imbalance in this relationship, a stress situation is generated. This can trigger worker feelings of job dissatisfaction. Conversely, psychological load is decreased and job satisfaction increases when organizational incentives are aligned with the workers’ expectations (Ovadje, 2009).

The dimension “reward” of ERI not only assesses financial gains, but also refers to feelings of recognition and respect at the work environment, i.e., expectations of job promotion, compatibility of the position occupied with worker’s educational level, and the feeling of fair relationships. The relevance of this dimension in the studied population was verified, with a positive association between low reward and work dissatisfaction, although it has not reached statistical significance levels.

A study conducted with Family Health Program professionals found that different realities, affection, tenderness, reliability, and gratitude on the part of patients were aspects related to the satisfaction (Fadel et al., 2008). These forms of rewards achieved in health work may not have been adequately captured by ERI. Rewards may mostly come from conviviality with staff and patients, along with the sense of usefulness of the work developed.

Introducing the dimension “overcommitment” in the effort-reward model has increased the power of association with the outcome analyzed. This dimension can be defined as a set of attitudes, behaviors, and emotions that result in excessive effort, along with the need for recognition and approval. Exaggeration in the labor efforts may contribute to the unbalance of the effort-reward relationship.

After adjusting the confounding variables, the measures of association for the combined model exceeded the prevalence ratios of the partial and full models. This has confirmed a better performance of combined models for investigating the outcomes among health workers (Araújo et al., 2016; Griep et al., 2011; Pinhatti et al., 2018). As the DC and ERI models measure different psychosocial aspects of work, the combined model is empowered to better predict outcomes in the workers’ health (Yu et al., 2013).

It is worth mentioning that despite all the precautions taken to avoid biases, one must consider the biases and limitations inherent to cross-sectional studies such as reverse causality, memory bias, and healthy worker bias.

## Conclusion

This study identified that the psychosocial aspects of work evaluated by using different ERI and MDC models (partial, full, and combined) were positively associated with job dissatisfaction. This conclusion indicates need for changes and improvements at work organization/environment, with the purpose of reducing stress and increasing levels of satisfaction. The full DC and ERI models performed better than the partial ones (dimensions assessed separately), which showed the relevance of the dimensions “social support” and “overcommitment” to job dissatisfaction. The DCM presented greater measures of association than ERI. However, the models combined were demonstrably advantageous to investigate job dissatisfaction levels, with more prevalence ratios than the isolated models (partial and full ones) after adjusting the confounding variables.

Finally, considering the complexity of occupational stress and the difficulty of its evaluation, the use of more advanced methods of analysis can lead to important gains for public health as combined models perform better for detecting events related to workers’ health.

## Data Availability

The datasets used and/or analyzed during the current study are available from the corresponding author on reasonable request.

## Abbreviations

CI: Confidence interval
DC: Demand-control
DCM: Demand-control model
ERI: Effort-reward imbalance
ILO: International Labour Office
OC: Overcommitment
PR: Prevalence patio
